# Comorbidities treated in primary care in children with chronic fatigue syndrome / myalgic encephalomyelitis: A nationwide registry linkage study from Norway

**DOI:** 10.1186/s12875-016-0527-7

**Published:** 2016-09-02

**Authors:** Inger J. Bakken, Kari Tveito, Kari M. Aaberg, Sara Ghaderi, Nina Gunnes, Lill Trogstad, Per Magnus, Camilla Stoltenberg, Siri E. Håberg

**Affiliations:** 1 Norwegian Institute of Public Health, PO Box 4404, Nydalen, Oslo 0403 Norway; 2 The National Center for Epilepsy, Oslo University Hospital, Oslo, Norway; 3 Department of Global Public Health and Primary Care, University of Bergen, Bergen, Norway

**Keywords:** Children, Adolescents, Chronic fatigue syndrome, Diabetes, Infections diseases, Multimorbidity, Primary care, Epidemiology

## Abstract

**Background:**

Chronic fatigue syndrome/myalgic encephalomyelitis (CFS/ME) is a complex condition. Causal factors are not established, although underlying psychological or immunological susceptibility has been proposed. We studied primary care diagnoses for children with CFS/ME, with children with another hospital diagnosis (type 1 diabetes mellitus [T1DM]) and the general child population as comparison groups.

**Methods:**

All Norwegian children born 1992–2012 constituted the study sample. Children with CFS/ME (*n* = 1670) or T1DM (*n* = 4937) were identified in the Norwegian Patient Register (NPR) (2008-2014). Children without either diagnosis constituted the general child population comparison group (*n* = 1337508). We obtained information on primary care diagnoses from the Norwegian Directorate of Health. For each primary care diagnosis, the proportion and 99 % confidence interval (CI) within the three groups was calculated, adjusted for sex and age by direct standardization.

**Results:**

Children with CFS/ME were more often registered with a primary care diagnosis of weakness/general tiredness (89.9 % [99 % CI 88.0 to 91.8 %]) than children in either comparison group (T1DM: 14.5 % [99 % CI: 13.1 to 16.0 %], general child population: 11.1 % [99 % CI: 11.0 to 11.2 %]). Also, depressive disorder and anxiety disorder were more common in the CFS/ME group, as were migraine, muscle pain, and infections. In the 2 year period prior to the diagnoses, infectious mononucleosis was registered for 11.1 % (99 % CI 9.1 to 13.1 %) of children with CFS/ME and for 0.5 % (99 % CI (0.2 to 0.8 %) of children with T1DM. Of children with CFS/ME, 74.6 % (1292/1670) were registered with a prior primary care diagnosis of weakness / general tiredness. The time span from the first primary care diagnosis of weakness / general tiredness to the specialist health care diagnosis of CFS/ME was 1 year or longer for 47.8 %.

**Conclusions:**

This large nationwide registry linkage study confirms that the clinical picture in CFS/ME is complex. Children with CFS/ME were frequently diagnosed with infections, supporting the hypothesis that infections may be involved in the causal pathway. The long time span often observed from the first diagnosis of weakness / general tiredness to the diagnosis of CFS/ME might indicate that the treatment of these patients is sometimes not optimal.

## Background

Chronic fatigue syndrome (CFS), or myalgic encephalomyelitis (ME), is a debilitating disorder, characterized by severe fatigue of unknown cause and often accompanied by other symptoms such as muscle pain and headache [1, 2]. The terms CFS and ME are often used interchangeably, and Norwegian health authorities recommend using the combined term CFS/ME [3]. In addition to fatigue, CFS/ME is associated with symptoms such as post-exertional malaise, pain, unrefreshing sleep, and cognitive impairment [1]. We have previously shown that CFS/ME is threefold more common among women than among men, with highest incidence rates among children and adolescents aged 10 to 19 years [4].

In Norway, children with possible CFS/ME are routinely referred by their general practitioner to hospital for diagnostic workup, while follow-up after diagnosis is the responsibility of the general practitioner. In the present population-based registry study we have used data from Norwegian primary and specialist health care registries with national coverage to further investigate the burden of CFS/ME in children. The study had two main objectives. Our first aim was to describe comorbidities diagnosed in primary care in children diagnosed with CFS/ME in specialist health care with children in the general population as control group. As treatment-seeking study-samples may be different from the general population in terms of registered comorbidities, we also used a comparison group of children with a chronic disease, type 1 diabetes mellitus (T1DM). Our second aim was to describe the timing of the diagnoses from primary care in relation to the timing of the CFS/ME diagnosis.

## Methods

### Study setting

Norway has a nationwide public health care system. Access to specialists requires referral from a general practitioner. The system is financed through governmental funding, and health care is free of charge for children up to the age of 16 years. Outpatients aged 16 years and up pay a minor fee, while all hospitalizations are free of charge.

### Data sources and definition of study groups

We linked data from national registries and health databases by using the unique 11-digit personal identification number provided to all Norwegian residents.

Children with CFS/ME and children with T1DM were identified through the Norwegian Patient Register (NPR) [5]. This registry contains data from all Norwegian hospitals and outpatient clinics. Reporting to the NPR is mandatory and is linked to the reimbursement system. Discharge diagnoses are reported as International Classification of Disease, Version 10 (ICD-10) codes. Administrative data reported include the patient’s personal identification number and dates for hospitalization and discharge.

The Norwegian Directorate of Health reimburses consultations in general practice. Diagnoses are reported as International Classification of Primary Care, Second Edition (ICPC-2) codes, together with the patient’s personal identification number and the date of consultation. We had access to information on a range of ICPC-2 codes for all Norwegian residents for the time period 2006–2014.

The study population was identified by using data from the National Registry and included children and adolescents (hereafter referred to as “children”) resident in Norway during 2008–2014. The children were followed through the calendar year they turned 18 years. To allow for a follow-up window of at least 2 years, we included children who were born between 1992 and 2012 to the study population.

**Fig. 1:**
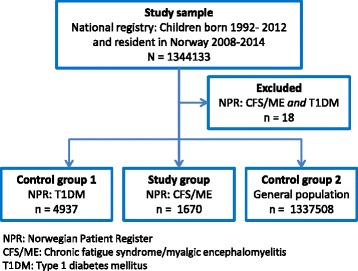
Study flow chart. A flow diagram illustrating the selection of the study groups

Children were defined as having CFS/ME if they were registered as in- or outpatients in Norwegian hospitals with the ICD-10 code G93.3 (“postviral fatigue syndrome/benign myalgic encephalomyelitis”). Similarly, children were defined as having T1DM if they were registered as in- or outpatients in Norwegian hospitals with ICD-10 code E10 (“type 1 diabetes mellitus”). In the total study sample of 1344133 children born 1992–2012 and resident in Norway during 2008–2014, we identified 1670 children with CFS/ME but not T1DM and 4937 children with T1DM but not CFS/ME (Fig. 1). A total of 18 children registered with both CFS/ME and T1DM were excluded from the study population. This left 1 337 508 children not registered with CFS/ME or T1DM before the age of 18 years for the general child population control group.

### Statistical analysis

For each ICPC-2 diagnosis, the proportion of children registered was calculated in each group. Proportions were adjusted for sex and age by direct standardization, using the CSF/ME group as the standard population. Non-overlapping confidence intervals (99 %) show statistically significant differences in proportions. With the exception of the ICPC-2 codes for diabetes (T89/T90), only ICPC-2 codes registered for more than 50 patients with CFS/ME were included to the analyses.

The Stata software package, Version 13.1 (StataCorp. 2013. *Stata Statistical Software: Release 13*. College Station, TX, USA: StataCorp LP) was used for data analysis.

## Results

The mean age at CFS/ME diagnosis among the 1670 children registered at least once in specialist health care with this diagnosis was 14.8 years (standard deviation [SD] 2.5 years) and 66.7 % were girls. Mean age at first diagnosis among the 4937 children in the T1DM control group was 10.9 years (SD 4.2 years) and 46.5 % were girls. The general child population control group consisted of 1 337 508 children (48.7 % girls).

### Diagnoses from primary care

Table 1 shows the proportions of children in each group registered with the primary care diagnoses.

**Table 1:** Proportion registered with primary care diagnoses among children with CFS/ME or type 1 diabetes mellitus (T1DM) in specialist health care, and for children in the general population

	CSF/ME (*n* = 1670)		T1DM (*n* = 4937)		General child population (*n* = 1 337 508)	
	# with condition	Proportion % (99 % CI)	# with condition	Proportion % (99 % CI)	# with condition	Proportion % (99 % CI)
*Diagnoses related to CFS/ME*						
Weakness / general tiredness (A04)	1501	89.9 (88.0–91.8)	666	14.5 (13.1–16.0)	140 915	11.1 (11.0–11.2)
Neurasthenia (P78)	156	9.3 (7.5–11.2)	2	0.0 (0.0–0.1)	1220	0.1 (0.1–0.1)
Sleep disturbance (P06)	258	15.4 (13.2–17.7)	204	4.1 (3.3–4.9)	43 795	3.3 (3.2–3.3)
Muscle pain (L18)	159	9.5 (7.7–11.4)	173	3.8 (3.0–4.6)	29 242	2.3 (2.3–2.4)
*Common mental disorders*						
Depressive disorder (P76)	230	13.8 (11.6–15.9)	231	5.4 (4.4–6.3)	32 399	2.7 (2.7–2.8)
Anxiety disorder/anxiety state (P74)	73	4.4 (3.1–5.7)	83	1.8 (1.3–2.4)	13 347	1.1 (1.1–1.1)
*Diagnoses related to infections*						
Influenza (R80)	412	24.7 (22.0–27.4)	739	15.1 (13.6–16.5)	133 281	10.1 (10.0–10.1)
Infectious mononucleosis (A75)	288	17.2 (14.9–19.6)	164	3.7 (2.9–4.5)	35 662	2.9 (2.8–2.9)
Tonsillitis acute (R76)	313	18.7 (16.3–21.2)	577	12.3 (11.0–13.6)	162 526	12.3 (12.3–12.4)
Strep throat (R72)	236	14.1 (11.9–16.3)	483	9.9 (8.7–11.0)	126 394	9.6 (9.5–9.6)
Pneumonia (R81)	207	12.4 (10.8–14.5)	356	7.3 (6.2–8.3)	90 027	6.7 (6.6–6.7)
*Other diagnoses*						
Diabetes (T89/T90)	1	0.1 (0.0–0.2)	4238	86.0 (84.6–87.4)	1079	0.1 (0.1–0.1)
Asthma (R96)	292	17.5 (15.1–19.9)	579	11.3 (10.1–12.6)	161 103	11.5 (11.4–11.6)
Migraine (N89)	187	11.2 (9.2–13.2)	128	3.0 (2.3–3.7)	30 255	2.5 (2.5–2.5)
Hyperkinetic disorder (P81)	54	3.2 (2.1–4.3)	175	3.0 (2.4–3.6)	37 387	2.4 (2.4–2.5)
Congenital anomaly musculoskeletal (L82)	54	3.2 (2.1–4.3)	89	1.8 (1.3–2.3)	28 096	2.0 (2.0–2.1)
Child behaviour symptom/complaint (P22)	71	4.3 (3.0–5.5)	143	2.7 (2.1–3.4)	37 241	2.5 (2.5–2.5)
Psychological disorders, other (P99)	55	3.3 (2.2–4.4)	95	1.9 (1.3–2.4)	17 008	1.2 (1.2–1.2)
Endocrine/metabolic/nutritional disorder, other (T99)	65	3.9 (2.7–5.1)	152	3.4 (2.7–4.2)	21 051	1.7 (1.7–1.7)

Proportions were adjusted for sex and age group (3-year categories) by direct standardization, using the population with CFS/ME as the reference population

Among children with CFS/ME, the most frequently observed primary care diagnosis was “weakness / general tiredness” (ICPC-2 code A04). This diagnosis was registered for 89.9 % of children in the CFS/ME group, 14.5 % in the T1DM group, and 11.1 % in the general child population group. “Neurasthenia” (ICPC-2 code P78) and “sleep disturbance” (ICPC-2 code P06) are other codes that are closely related to CFS/ME. Neurasthenia was hardly ever used as a diagnosis for children in the two control groups, while 9.3 % of the children in the CFS/ME group had this diagnosis. Sleep disturbance was also far more frequent in the CFS/ME group, as was muscle pain (CFS/ME: 9.5 %, T1DM: 3.8 %, general child population: 2.3 %).

Furthermore, we found higher frequencies of depression and anxiety in the CFS/ME group (13.8 and 4.4 %, respectively) than in the T1DM (corresponding figures: 5.4 and 1.8 %) and general child population control groups (corresponding figures: 2.7 and 1.1 %).

Elevated frequencies of all diagnoses related to infection were observed in the CFS/ME group. In particular, infectious mononucleosis was far more frequent in this group (17.2 %) than in the control groups (T1DM: 3.7 %, general child population: 2.9 %). Influenza, acute tonsillitis, “strep throat”, and pneumonia were also more frequent in the CFS/ME group.

While ICPC-2 codes for diabetes were registered for 86.0 % of children in the T1DM group, these codes were rarely registered for children without a specialist health care diagnosis of T1DM (CFS/ME group and general child population).

Asthma was also more common in the CFS/ME group (17.5 %) than in the two control groups (11 % in both groups).

### Timing of the diagnoses

Comparing only primary care diagnoses within the 2 years prior to the specialist health care diagnoses revealed even stronger differences between the CFS/ME group and the T1DM group (Table 2).

**Table 2:** Proportion registered with primary care diagnoses within the period 2 years prior to the first registered CFS/ME or type 1 diabetes mellitus (T1DM) diagnosis in specialist health care

	CSF/ME (*n* = 1670)		T1DM (*n* = 4937)	
	# with condition	Proportion % (99 % CI)	# with condition	Proportion % (99 % CI)
*Diagnoses related to CFS/ME*				
Weakness / general tiredness (A04)	1226	73.4 (70.6–76.2)	164	3.5 (2.8–4.3)
Neurasthenia (P78)	58	3.5 (2.3–4.6)	1	0.0 (0.0–0.1)
Sleep disturbance (P06)	94	5.6 (4.2–7.1)	42	0.9 (0.5–1.2)
Muscle pain (L18)	59	3.5 (2.4–4.7)	37	0.8 (0.4–1.2)
*Common mental disorders*				
Depressive disorder (P76)	94	5.6 (4.2–7.1)	19	0.4 (0.1–0.6)
Anxiety disorder/anxiety state (P74)	19	1.1 (0.5–1.8)	15	0.3 (0.1–0.5)
*Diagnoses related to infections*				
Influenza (R80)	173	10.4 (8.4–12.3)	154	3.1 (2.4–3.8)
Infectious mononucleosis (A75)	185	11.1 (9.1–13.1)	25	0.5 (0.2–0.8)
Tonsillitis acute (R76)	85	5.1 (3.7–6.5)	161	3.4 (2.6–4.1)
Strep throat (R72)	74	4.4 (3.1–5.7)	123	2.5 (1.8–3.1)
Pneumonia (R81)	83	5.0 (3.6–6.3)	107	2.2 (1.6–2.8)
*Other diagnoses*				
Diabetes (T89/T90)	0	0.0 (0.0–0.0)	1 362	28.2 (26.4–29.9)
Asthma (R96)	134	8.0 (6.3–9.7)	253	5.0 (4.1–5.8)
Migraine (N89)	102	6.1 (4.6–7.6)	30	0.6 (0.3–0.8)
Hyperkinetic disorder (P81)	35	2.1 (1.2–3.0)	73	1.2 (0.8–1.6)
Congenital anomaly musculoskeletal (L82)	19	1.1 (0.5–1.8)	32	0.7 (0.3–1.0)
Child behaviour symptom/complaint (P22)	32	1.9 (1.1–2.8)	52	1.0 (0.6–1.4)
Psychological disorders, other (P99)	22	1.3 (0.6–2.0)	17	0.3 (0.1–0.5)
Endocrine/metabolic/nutritional disorder, other (T99)	18	1.1 (0.4–1.7)	54	1.3 (0.8–1.7)

Proportions were adjusted for sex and age group (3-year categories) by direct standardization, using the population with CFS/ME as the reference population

For instance, while 73.4 % of the CFS/ME patients had been diagnosed with weakness / general tiredness prior to their CFS/ME diagnosis, this was the case for only 3.5 % of T1DM patients. Similar results were found for muscle pain, depression, anxiety, and migraine. Also, strong differences were found between the CFS/ME group and the T1DM group for diagnoses related to infection. While 11.1 % of children with CFS/ME had been diagnosed with infectious mononucleosis prior to the specialist health care diagnosis, this was the case for 0.6 % of children with T1DM. Other diagnoses related to infections (influenza, acute tonsillitis, “strep throat” and pneumonia) were 2–3 times more common in the CFS/ME group than in the T1DM group.

### Time from first diagnosis of weakness / general tiredness to CFS/ME diagnosis

Among the 1670 children with CFS/ME, 1292 (74.6 %) were registered with at least one prior primary care diagnosis of weakness / general tiredness (follow-up 2006–2014). A total of 782 (46.8 %) were registered at least four times with this diagnosis. For a large proportion of children with a prior weakness / general tiredness diagnosis, the time span to the CFS/ME diagnosis was 1 year or longer (617/1292, 47.8 %).

## Discussion

In this large, population-based study, children and adolescents with CFS/ME were significantly more often diagnosed with several conditions in primary care than children with T1DM and children in the general population. These diagnoses not only included conditions closely related to CFS/ME but also depression, anxiety, infections, and migraine. When restricting to primary care diagnoses prior to the specialist health care diagnoses, even stronger differences between children with CFS/ME and children with T1DM were observed. Three out of four children with CFS/ME in specialist health care were registered with a prior diagnosis of weakness / general tiredness in primary care. However, as we had access to specialist health care diagnoses for the period 2008–2014 and primary care diagnoses only from 2006 to 2014, it cannot be ruled out that the proportion with this diagnosis in primary care was even higher. For many children the time span from the first primary care diagnosis of weakness / general tiredness to the first CFS/ME diagnosis was 1 year or longer.

CFS/ME is a rare disease and previous studies have typically included small number of patients. The main strength of this study is the utilization of registry data covering the entire population over a long study period. Children with a serious condition of prolonged nature (in this case CFS/ME) are likely to be followed up more closely in primary care than children without such a condition. By including a control group with another chronic condition (T1DM) we were able to make comparisons of children with CFS/ME both to the general child population and to a chronic condition group.

As in other studies based on observational, routinely collected data, a major limitation is the lack of validity testing of the diagnoses. While ICD-10 code G93.3 is to be used for CFS/ME in specialist health care, there is no specific ICPC-2 code for the condition that is to be used in primary care. The observation that children diagnosed with CFS/ME in specialist health care were far more often registered with the ICPC-2 code for weakness / general tiredness in primary care than children with T1DM and children in the general child population is reassuring with regard to the reliability of the CFS/ME diagnosis from specialist health care. Similarly, ICPC-2 codes for diabetes were registered in primary care for most children with the ICD-10 code for T1DM in specialist health care, but only for a small proportion of the children in the two other groups. The Norwegian Directorate of Health recommends use of the ICD-10 code G93.3 for CFS/ME [3]. Still, some children fulfilling criteria for a diagnosis of CFS/ME might have been registered with other ICD-10 codes. In a recent study, paediatricians generally reported that they follow the guidelines of the Norwegian Paediatric Association [6].

In the present study we found a high frequency of being diagnosed with depression among children with CFS/ME. In a recent case-control study from Norway, children (12–18 years of age) with CFS/ME according to strict clinical criteria had a strong reduction in health-related quality of life compared to healthy peers [7]. That study also reported that children with CFS/ME had eight times higher risk of depressive symptoms, but that this difference could not explain their poor quality-of-life scores. High levels of depression (29 %) have also been reported from a large UK cross-sectional study among children aged 12–18 years with CFS/ME [8]. We found higher frequencies of anxiety diagnoses among children with CFS/ME than in our two control groups. Another study from the UK included children with CFS/ME 7–17 years of age and compared anxiety scores for this group to normal population scores [9].

In a previous study using data from the NPR 2008–2012, we have shown that there are two distinct age peaks in the incidence of CFS/ME (age groups 10 to 19 years and 30 to 39 years) [4]. We hypothesized that this pattern could be explained by primary exposure to infectious agent(s) in adolescents (first age peak) and subsequent reactivation of latent infection (second age peak). In the present study, we have shown that the frequency of infections diagnosed in primary care is higher prior to a diagnosis of CFS/ME than prior to a diagnosis of T1DM, indicating that infections may indeed be among the factors that could cause CFS/ME. In a recent review, it was concluded that the severe fatigue that can afflict patients with a disorders such as multiple sclerosis, Parkinson’s disease, and systemic lupus erythematosus, may be caused by peripheral inflammation and immune activation, and that similar mechanisms may also play a role in the development of CFS/ME [10]. In a cohort of 301 adolescents with infectious mononucleosis from Chicago, Illinois, 13 % met the criteria for chronic fatigue syndrome after 6 months [11]. Also, in an adult UK cohort consisting of patients with infectious mononucleosis, influenza or tonsillitis (but no control group without infection), the odds of clinically diagnosed fatigue was found to be four times higher for infectious mononucleosis when compared to influenza and nearly seven times higher for infectious mononucleosis when compared to tonsillitis [12]. These results fit well with results from the present study, where we observed that the greatest difference between the CFS/ME group and either control group was for infectious mononucleosis. However, influenza, acute tonsillitis, strep throat, and pneumonia were also significantly more often observed in the CFS/ME group, indicating that different infections might trigger the development of CFS/ME. In a previous study we found a strong, positive association between pandemic influenza A (H1N1) and CFS/ME, but no changes in risk of CFS/ME after pandemic influenza vaccination [13]. Similarly, an earlier Norwegian study showed no relation between vaccination against meningococcal disease and CFS/ME, while a tenfold increased risk for CFS/ME was observed after mononucleosis with clear symptoms [14].

We found a somewhat higher frequency of asthma among children with CFS/ME than in the T1DM and general child population control groups. In US adults, high rates of asthma have been described prior to the onset of CFS [15], and from Taiwan, the risk has been reported to increase following atopy [16]. We also observed that one in ten patients with CFS/ME had been diagnosed with migraine in primary care, which was four times as frequent as in patients with T1DM. The link between CFS/ME and migraine seems rather well established. For instance, a 1.5-fold increased risk of CFS was described in adults with migraine in another study from Taiwan [17]. Also, in a study including two different adult groups of patients with CFS, migraines were often reported (39–69 %) [18]. Similar to our findings, high frequencies of headaches and muscle pain were observed among children (mean age 15.2 years) with CFS/ME in Dutch paediatric departments [19].

## Conclusion

The findings in the present study confirm results from previous studies in that children with CFS/ME show higher levels of depression, anxiety, migraine, and muscle pain than their peers. Furthermore, children with CFS/ME are often diagnosed with infections, in particular prior to the CFS/ME diagnosis. The long time spans observed from the first diagnosis of weakness / general tiredness in primary care to a specialist health care diagnosis of CFS/ME might indicate that the treatment of these patients is sometimes not optimal.

## Abbreviations

CFS: Chronic fatigue syndrome
CI: Confidence interval
ICD-10: International Classification of Disease, Version 10
ICPC-2: International Classification of Primary Care, Second Edition
ME: Myalgic encephalomyelitis
NPR: Norwegian Patient Register
T1DM: Type 1 diabetes mellitus
